# The Pigmentary Syphiloderm

**Published:** 1878-10

**Authors:** I. Edmonson Atkinson

**Affiliations:** Physician to the Baltimore Special Dispensary


### THE PIGMENTARY SYPHILODERM.

BY I. EDMONDSON ATKINSON, M. D.,
PHYSICIAN TO THE BALTIMORE SPECIAL DISPENSARY.

(*Read before the American Dermatological Association, at Saratoga Springs, N. Y., Aug. 27th*, 1878.

Before presenting the cases whose histories form the basis of this paper, I may be pardoned for briefly reviewing the characters of the lesion of which I desire to speak.

In describing "syphilide pigmentaire" for the first time in 1853, Hardy ascribed to it peculiarities separating it from all other syphilodermata upon the one hand, and upon the other from the various forms of non-specific pigmentary abnormalities of the skin. The very lucid and faithful account of the symptoms of this lesion given by this author, has been supplemented by other writers, but has more especially received support from the pen of Fournier (Leçons sur la Syphilis etudiés plus particulièrement, chez la Femme, 1873, p. 422). According to these authors, the pigmentary syphiloderm may appear from the fourth month to the end of the second year of the disease, and is consequently one of its later secondary manifestations. It is observed almost exclusively in the female sex, occupying preferably the skin of the neck (29 times in 30, Fournier), although occasionally met with upon other portions of the body. Its symptoms are, briefly, as follows: Upon the neck, more commonly upon its lateral surfaces, appear, with or without concomitant symptoms, but invariably subsequent to other manifestations of general syphilis of other parts of the body (roseola, papules, mucous patches, adenopathies, etc.), faintly colored spots, varying in size from that of a split pea to that of a finger nail, which rarely remain discrete, but rather run together, being connected by more or less narrow bands of similar discoloration, and forming an extent of pigmented surface resembling a clumsy network whose meshes are represented by areas of normal skin, or following Hardy and others, of skin whiter than normal (Leçons sur la Scrofule et les Scrofulides et sur la Syphilis et les Syphilides, 1864, p. 175). The color of the pigmented area is of a very faint grayish-yellow, a café-au-lait color, paler than that of tinea versicolor or than that of chloasma uterinum. Fournier compares the effect to the dirty necks of uncleanly people. It is uninfluenced by pressure.

The borders of the patches lose themselves more or less imperceptibly in the healthy skin. Their surface is smooth, absolutely without desquamation, not at all elevated above the general surface, giving rise to no subjective symptoms, and quite rebellious to treatment, whether anti-syphilitic or otherwise, enduring for months—it is even said for years.

Such, then, being a general outline of this lesion, I propose to offer the following cases as illustrating it, before proceeding to discuss the claims upon which it rests its position in the nosology of syphilis.

CASE 1. Hannah F., white, 17 years old, unmarried, of medium size and complexion inclining to brunette, with dark hair and eyes, came to the dispensary June 10th, 1875. She said that during the previous summer she had had suppurating buboes in both groins (the scars of which are at present visible). Shortly after this she had sores upon her vulva, and five months ago an eruption first came upon her skin. Upon examination, her labia majora and minora were found to be very œdematous and infiltrated from the irritation of numerous mucous patches, which also extended along the perineum. Over her thighs and abdomen were the stainings left by a previous syphilitic eruption. Mucous patches were present upon the faucial mucous membrane. There was a general adenopathy, especially marked in the cervical region. Distributed over the neck and shoulders, and extending over the deltoid region of the arms quite symmetrically, were, what appeared upon superficial examination to be spots of abnormally whitened integument, scattered irregularly and discretely over the surface. Upon more rigid examination, these spots, which did not exceed from six to twelve millimeters in diameter, were seen to be not whiter than normal, the true alteration being evidently in the surrounding portions of the skin. This alteration consisted of pigmented spots extending over the front and sides of the neck and shoulders, and connected with each other uninterruptedly, except where the patches of normal skin seemed to be inserted, forming the network-like appearance characteristic of the eruption. This maculation, which resembles in coloration a faint chloasma uterinum, is less pronounced than that of tinea versicolor, and also differs from that following the ordinary syphiloderms, being paler. The surface was perfectly smooth and free from desquamation. There was no itching. The patient was, however, much mortified at the dirty appearance of her skin, which, when first noticed by her a short while ago, was just as at present. She was of careful, cleanly habits, and was quite sure that there had been no alteration in the appearance of the eruption since it was first noticed. She was subjected to an appropriate mercurial internal and external treatment, which was followed with considerable regularity. Upon July 24th, it was noted that the treatment had effected the disappearance of all the mucous patches except one at the fourchette, and that the labia were becoming much less infiltrated. The maculation about the shoulders and neck persisted, though fainter. She was under treatment steadily until August 11th, the maculations becoming fainter, and general improvement being steadily experienced. She was not seen again until January 25th, 1876, when she informed me that she was married and three months pregnant. Upon examination, she appeared quite well, the infiltration of the labia having quite disappeared, as well as the maculations upon her neck and shoulder. She did not again come under observation.

Case 2.—June 19th, 1877, Becky H., a young prostitute, born in Pennsylvania, 19 years old, of moderately fair complexion, with blue eyes and light hair, and of healthy appearance, applied at the dispensary to be treated for a vaginal discharge of three weeks' duration. She said she had had a similar discharge during the previous January. Upon physical examination, the integument of her abdomen and chest anteriorly, and of her thighs, was well sprinkled with a fading roseola. There were no signs of an ulcer upon the genital organs, but there was a painless inguinal adenopathy. She was kept under close observation until July 7th. She re-applied October 23rd, complaining of pharyngeal mucous patches, but without any cutaneous eruption. December 8th. The mucous patches which had healed, had now returned. December 29th. Having now been at least seven months syphilitic, it was now noted that upon both sides of her neck, arranged with tolerable symmetry, were a number of faintly brownish spots, similar in coloration to those of the previous patient. They were limited to the sides of the neck, and did not *inclose* unpigmented areas, not being connected so as to form the usual network. She had been almost constantly under my observation, and neither she nor I had observed a previous eruption upon these parts. She quite decidedly denied that any such had been present. There were no subjective symptoms, and the spots were smooth, unelevated, and not desquamating. Their margins were not very well defined. By February 19th, the maculations were much fainter, but had undergone no change in distribution. March 3rd. There had been for several days a right iritis. The maculations had not diminished in size or configuration ; they had simply faded, and more nearly approached the normal hue of the skin. During all this period, the patient was almost constantly undergoing a mercurial treatment. She has not again come under observation.

My third case was an anomalous one in several particulars, and I have hesitated before including it with cases of pigmentary syphiloderm, having never seen a similar condition ; but upon reflection I am unable to assign it to any other form of syphilitic eruption than the one under consideration. It is as follows, viz. :

Case 3.—Annie J. came to the dispensary October 10th, 1875. She was a light mulatto, 18 years of age, and of a moderately healthy appearance. At the posterior commissure of the labia minora there was a soft spreading ulcer, accompanied by indolent inguinal adenopathy. She had, also, some sore throat, enlargement of the cervical glands, and complained of pains in the arms and sides. No reliable history could be obtained, although it was evident that the lesions were of some duration. The sore was cauterized with the acid nitrate of mercury. She did not again appear at the dispensary until March 28th, 1876, five months after her first appearance. She then said that her eyes had been troubling her for about two months. This was found to be due to a double iritis ; and there was a small perforation of the left cornea, through which the iris slightly protruded. Upon the thighs was a fading papular eruption. Upon the dorsal surfaces of the hands and wrists, and upon the inner and extensor surfaces of the knees, were very symmetrically arranged mottled appearances, resembling, when seen at a distance, old scars from burns, but really due to pigmentary changes alone. Accôrding to her, they had been present nearly all winter. As in my first case, my first impression was that the lighter-colored islets were the affected portions, but a moment's careful examination served to correct this error. These areas, which varied in size from five to fifteen millimeters in diameter, were of the color of the normal skin, and only appeared lighter by contrast with the surrounding darker surface, which was almost black, and extended continuously, the different spots being connected by narrow bands, and forming the lace-like arrangement already spoken of. The borders were abruptly margined. The normal café-au-lait color of the general surface contrasted strikingly with the hyper-pigmented portions, the general effect seemingly justifying the term piebald in describing it. No other portion of the skin was similarly affected. She was last seen May 31st, when, after taking cod liver oil and the syrup of the iodide of iron, she was much better. The maculations had faded from her hands, but were still present upon the inner surfaces of both knees.

The pigmentary changes of which the foregoing cases afford examples are so unfrequently observed that many writers have been led to deny that they are to be considered direct results of syphilis. The insignificance of the symptoms, and the probability of its being frequently overlooked must, however, be considered. Dr. G. H. Fox, who, however, regards the lesion as a vitiligo, very properly draws attention to this point (*Amer. Journ. Med. Sciences*, April, 1878), and thinks that it will much more frequently be observed, when searched for, even in the male sex. On the other hand, there is danger that there may be committed the error of including with the pigmentary, syphiloderm, lesions of variously different origins. These are principally the chloasmata, chloasma uterinum, and chloasma cachecticorum, and the stainings left behind by a roseola syphilitica. (It is hardly necessary here to refer to the danger of confounding tinea versicolor with the pigmentary syphiloderm, since the points of a differential diagnosis are always at hand in the presence of a parasite in the desquamated epidermis of the former.) The ordinary chloasmata may usually be distinguished by attention to their color, distribution and configuration, as well as to the constitutional conditions of the patients. Their color is usually darker; they are more uniform in their distribution, not inclosing the mesh-like spaces, and are most rarely confined to the neck, the favorite seat of the lesion under discussion. (The chloasma that is seen frequently in old syphilitics, is simply due to the cachexia, and is altogether unlike the truly syphilitic form.)

The stainings left behind by a syphilitic roseola, to my mind, more nearly approach, in their characteristics, the pigmentary syphiloderm, than any other form of lesion. It is not at all uncommon to see upon the abdomen especially, tracts of hyper-pigmented skin inclosing circular areas of normal color. These tracts are the remains of syphilitic roseola; but here, however, there is usually the clear history of a preceding hyperæmia, the hyper-pigmentation is much more extensive, and its duration much less prolonged. The especial point of distinction, however, is that the pigmentary syphiloderm appears independently of any preceding hyperæmia, according to the almost universal opinion of those who have observed it, and to the unanimous opinion of the patients. Until convincing clinical proof of the necessary pre-existence of this hyperæmia is produced, those who believe in the reality of this syphiloderm are entitled to hold to their faith.

There are others who, recognizing the fact that the pigmentary deposits are not due to preceding hyperæmia, consider the non-pigmented areas, or, as they claim, the whitened patches, to be the seat of the true morbid process, and that the lesion is a pigmentary atrophy of the inclosed spaces, around which there is an increased deposit of pigment, as is usually the case in acquired leucoderma.

It is true that some writers, as Hardy and Drysdale, while considering the hyper-pigmentation to be the essential lesion, also think that these inclosed spaces contain less coloring matter than normal ; and admitting that this occasionally is the case, it becomes possible, in a narrow sense, to designate such condition as leucodermatous. But in doing this we simply beg the question, since the transient and always very limited blanching, is invariably accompanied by a hyper-pigmentation always equaling, almost always exceeding, it in extent, and which has at least equal claims to be considered the essential lesion. I think it especially unfortunate that Dr. George H. Fox, who has most ably expressed his view of the leucodermatous nature of this affection, should have used the term vitiligo to designate it, a term that has been pretty generally applied to that form of lesion where there is a permanent, complete and usually progressive centrifugal disappearance of cutaneous pigment. It is true that Dr. Fox is unwilling that vitiligo should be thus restricted in its meaning, but nevertheless, in describing it, he quotes Dr. Duhring's definition of the condition, which certainly has reference to a definite, progressive and permanent loss of pigment.

It may be properly objected to the view that the supposed leucodermic patches occupy the sites of previous hyperæmic eruptions, that it is the positive opinion of other writers, based upon their personal experience, that the pigmentary syphiloderm as observed by *them*, has never succeeded upon the site of an antecedent eruption. The leucodermatous condition, moreover, is certainly not present in all cases, and it is a fact, as observed by Hardy, and evidenced in my second case, that the hyper-pigmented spots may occur without including islets of normal or whitened integument.

The resistance offered by this lesion to the influence of anti-syphilitic remedies, undoubtedly affords their most efficient weapon to those who combat the doctrine of its syphilitic nature. It is, for example, argued that all syphilitic alteration, especially those of the earlier constitutional periods, are promptly antagonized by the administration of mercury, and that the hyper-pigmentation under discussion, not being modified by this drug, cannot be a syphilitic manifestation. Such a conclusion is based upon incorrect ideas of the nature of the pigmentation. The pigmentary changes following the earliest syphilitic symptoms are always due to the pre-existing hyperæmia—their duration and intensity are usually proportioned to the duration and character of the hyperæmia, and they are principally deposited in the connective tissue of the corium, in the region of the vascular distribution. The rationale of pigmentation is too little known, but we do know that pigment disappears readily after the recession of a hyperæmia of short duration, while after an old eczema or chronic ulcer, it may remain for many years. Why this is so, is not known. It is a matter of universal clinical experience that the pigmentations following the early syphilitic eruptions are but little disposed to become permanent, and are readily re-absorbed. With the pigment substance of the epidermis, on the contrary, the case is different. Here the hyper-pigmentation may readily occur quite independently of hyperæmia, and is due to an increase of the melarin, the physiological pigment of the skin.

It is a matter of general experience that the non-vascular hyper-pigmentations are provokingly obstinate, and beyond the reach of internal medication. No one can explain why the different cachexiæ, or why uterine disorder should induce a hyper-pigmentation in the rete malpighii. There is no reason why the syphilitic dyscrasia, as distinct from any cachectic condition, may not equally evoke similar pigmentary changes, and with the light thrown upon the subject from time to time by corroborative cases, I have no hesitation in saying that it does evoke them, and venture to suggest that they may be amenable to the same therapeutic agencies.

